# Motor skill competence and moderate- and vigorous-intensity physical activity: a linear and non-linear cross-sectional analysis of eight pooled trials

**DOI:** 10.1186/s12966-023-01546-7

**Published:** 2024-02-07

**Authors:** L. M. Barnett, S. J. J. M. Verswijveren, B. Colvin, D. R. Lubans, R. M. Telford, N. J. Lander, N. Schott, M. Tietjens, K. D. Hesketh, P. J. Morgan, T. Hinkley, K. L. Downing, R. D. Telford, K. E. Cohen, N. D. Ridgers, G. Abbott

**Affiliations:** 1 Institute for Physical Activity and Nutrition (IPAN), School of Exercise and Nutrition Sciences, Deakin University, 221 Burwood Hwy, Burwood, 3125 Australia; 2 Institute for Physical Activity and Nutrition (IPAN), School of Health and Social Development, Deakin University, 221 Burwood Hwy, Burwood, VIC 3125 Australia; 3 School of Psychology, Deakin University, 221 Burwood Hwy, Burwood, VIC 3125 Australia; 4 Centre for Active Living and Learning, College of Human and Social Futures, University of Newcastle, University Drive, Callaghan, NSW 2308 Australia; 5 Faculty of Sport and Health Sciences, University of Jyväskylä, Keskussairaalantie 4, 40600 Jyväskylä, Finland; 6 University of Canberra, Research Institute for Sport and Exercise, Bruce, ACT 2617 Australia; 7 The Australian National University, National Centre for Epidemiology and Population Health, ANU College of Health & Medicine, 62 Mills Rd, Acton, ACT 2601 Australia; 8 Department of Sport Psychology and Human Movement Sciences Organization, University of Stuttgart, Institute for Sport and Movement Science, Allmandring 28, Stuttgart, 70569 Germany; 9 University of Muenster, Institute of Sport and Exercise Sciences, Horstmarer Landweg 62 b, 48149 Münster, Germany; 10 Melbourne, Australia; 11 University of South Australia, Alliance for Research in Exercise, Nutrition and Activity, Allied Health and Human Performance, Frome Road, Adelaide, SA 5001 Australia

**Keywords:** Child, Object control, Locomotor, Accelerometer

## Abstract

**Background:**

Few studies have examined the relationship between motor skill competence and device-measured physical activity in large samples and none have used non-linear modelling. This study assessed the linear and non-linear associations between motor skill competence and physical activity in children using pooled data from eight studies.

**Methods:**

Cross-sectional ActiGraph accelerometer and motor skills competence data from 988 children (50.8% boys) aged 3–11 years were included. Total, object control and locomotor skill competence were assessed using the Test of Gross Motor Skill Development. Linear mixed models were fitted to examine linear associations between motor skill competence and physical activity. Then, restricted cubic splines models were used to assess potential non-linear relationships. Interactions by sex and age were assessed.

**Results:**

There was evidence of positive linear associations between total skill, and object control and locomotor skills, with moderate- and vigorous-intensity physical activity; however, the associations with total skill competence and object control better fitted a non-linear model. Non-linear models indicated associations were positive but relatively weak in the low to mid ranges of TGMD/object control scores but at high ranges (~ > 70 out of 100/ and ~ 35 out of 50) the association strength increased for both moderate- and vigorous-intensity physical activity. There were sex interactions for locomotor skills only, specifically for vigorous activity with boys having a stronger positive association than girls.

**Conclusions:**

There appears to be a threshold for object control skill proficiency that children need to reach to enhance their physical activity levels which provides support for a motor skill “proficiency barrier”. This provides a tangible benchmark for children to achieve in motor competence programs.

**Supplementary Information:**

The online version contains supplementary material available at 10.1186/s12966-023-01546-7.

## Background

Many children in the twenty-first century do not meet physical activity guidelines [1], are less fit than previous generations [2], and more likely to be children with overweight or obesity [3]. In 2008, Stodden and colleagues published a developmental conceptual model highlighting the importance of developing gross motor skill competence to support engagement in physical activity throughout childhood and beyond [4]. Motor competence is a broad construct and includes object control manipulation (e.g. catching a ball), locomotor (e.g. running and jumping), stability (e.g. balancing), and coordination skills [5]. Specifically, the aforementioned model [4] describes a process of cyclical engagement whereby participating in regular physical activity leads to better motor skill competence which encourages and facilitates children to keep participating in physical activities, thereby developing higher levels of health-related fitness [6] and better perceptions of their motor skill competence [4]. This developmental model purports that the strength of the association between the key health-related constructs, including gross motor skill competence and physical activity, will increase as a child ages [4]. The model is considered developmental as when children are young, they engage in physical activity and gain motor skill competence but then as they age and develop motor skill mastery becomes more important for their continued physical activity participation [4]. When children are young and still developing skill competence, associations with moderate- to vigorous-intensity physical activity would likely be weak, whereas in middle childhood and beyond, associations are likely to be stronger [4].

Whilst the pathway between motor skill competence and physical activity is therefore considered reciprocal, the pathway of interest for this investigation rests on the concept of a motor skill “proficiency barrier” to engagement in physical activity [7]. This concept, introduced in 1980, purported that motor development occurred in a sequential fashion starting in infancy with reflexes, followed by postural reactions, and then the acquisition of ‘fundamental motor skills’ (e.g., throwing, kicking, running, jumping) in early childhood. The next progression was gaining the skills needed for more specific physical contexts such as games and sports. Seefeldt hypothesized that there was a proficiency barrier between fundamental motor skill development (which starts in early childhood) and transitional skills (typically acquired during middle childhood through to adulthood) [7]. Subsequently, it was suggested that children who did not pass through this ‘barrier’ would be less likely to be able to sustain the moderate- to vigorous-intensity physical activity needed for participation in games and sports [8]. Non-linear modelling could help inform at what skill level this barrier might occur, yet few studies have moved beyond modelling linear relationships. Typical analysis of associations between continuous measures consider only linear relationships (i.e., where a unit change in an exposure variable is associated with a fixed increase/decrease in an outcome), yet it is often plausible and even likely that the true relationships are non-linear in nature with varying dose-responses (e.g., increasing or diminishing returns for increases in exposure). For example, one study in Portugal investigated the association between BMI and motor competence and hypothesized a curvilinear association expressed in an inverted parabolic form, with children with lower and higher body mass index showing poorer motor skill competence [9].

Multiple reviews in the last decade, with the first in 2010 [10], support that children with higher motor competence are likely to be of healthy weight [5, 11], have higher health-related fitness [5, 11], and be more physically active than their less skilled peers [5, 12–14]. However, a recent systematic review examined longitudinal and experimental evidence for physical activity, health-related fitness, weight status, and perceived motor competence in relation to actual motor skill competence over the last 5 years and refuted some of the past evidence [15]. While findings highlighted strong evidence for motor competent children being fitter and of healthy weight, the evidence for an association with physical activity was less clear [15]. Measurement complexities may explain the lack of convincing evidence for a positive association between motor competence and physical activity found in the review. More specifically, each construct within the broader umbrella of motor skill competence (e.g., object control) has variable measurement approaches (e.g., focused on the quality or the outcome of movement) [5, 16, 17]. In addition, these different aspects of motor skill development can relate differently to health-related outcomes [15, 18]. Similarly, the measurement of physical activity is also complex and potentially limiting due to the type of activity it may not capture. Physical activity can be operationalized in terms of intensity (light- to moderate- to vigorous-intensity physical activity), frequency, duration, and type, and can be measured subjectively or using device-based measures (e.g., accelerometers); with many methods within each of these broad approaches used to quantify free-living activity [19]. Finally, the developmental aspects of the model authored by Stodden et al. [4], in terms of how the direction of these pathways change as children age, could not be assessed in the afore mentioned review which focused on longitudinal and experimental evidence [15]. This was not only due to the complexity in physical activity measurement, but also complicated by longitudinal studies starting at different points in childhood and having different follow-up periods. As such, there exists a need to use the same measures for physical activity and motor competence to minimise the impact of measurement error on outcomes and improve our understanding of this association.

One can only develop appropriate intervention content and strategies if the underlying mechanisms for the interaction of motor competence and physical activity in boys and girls during childhood are understood. In preschool children (aged 3 to 5 years), a recent review noted small significant increases in motor competence and physical activity levels after intervention, yet only a few trials assessed both outcomes [20]. Recent Cochrane review evidence reports minimal effects for school-based interventions on physical activity (albeit depending on the intervention foci), with less than 1 min per day increase in daily moderate- to vigorous-intensity physical activity (based on 33 studies) [21]. Also, typically, even when both outcomes are assessed, change in one variable (e.g., motor skill competence) is typically not analysed in relation to change in the other variable (e.g., physical activity) - instead these variables are treated as independent outcomes [15]. This analysis practice further prohibits our understanding of how these variables interplay in children of different ages.

Therefore, the main purpose of our study was to assess the linear and non-linear associations between motor skill competence and physical activity for children from the ages of 3 to 12 years in a large cross-sectional sample. Since we needed a large sample where data had been collected with the same measures, we decided to pool data from similar studies from the same country. Based on the work of Seedfeldt [7, 8], we hypothesized that the association between motor competence and moderate- to vigorous-intensity physical activity would be stronger once a threshold of motor skill competence development was attained. Hence, a secondary aim was to identify if a threshold of skill competency could be identified.

## Methods

The ‘Physical Activity & Fundamental Movement Skills Data Pool’ combined physical activity, motor skill competence, and demographic data from eight Australian intervention, longitudinal, or cross-sectional studies (the collaborators with similar data who responded to our invitation). All studies received parental consent to collect device-based physical activity using ActiGraph accelerometers and motor skill competence using the Test of Gross Motor Development (TGMD) in children aged 3–12 years. All studies received ethical approval from their institution. Relevant to this study, an ethics application was approved for the pooling of data with agreements between Deakin University and each institution involved (2020–091 Deakin University Human Research Ethics Committee). The present manuscript is reported following the STROBE statement (Supplementary File 1, Table S1) [22].

### Participant information and demographics

The details of all individual studies used in the current data pooling project are reported in Table 1. The studies were: 1. Active Electronic Games and Motor Skills (*GamesSkill* [23, 24]), 2. Global Assessment of Children’s Motor Competence (*Global_MC* [25]), 3. Fitness, Activity, and Skills Testing (*FAST* [26]), 4. Healthy Active Preschool and Primary Years (*HAPPY* [27]), 5. Infant Feeding Activity and Nutrition Trial (*InFANT* [28]), 6. Physical Education and Physical Literacy (*PEPL* [29]), 7. Actual and Perceived Skill and Physical Activity (*SkillPA* [30, 31]), and 8. Supporting Children’s Outcomes using Rewards, Exercise and Skills (*SCORES* [32, 33]).

**Table 1:** Individual study characteristics of studies used in the ﻿‘Australian Physical Activity & Fundamental Movement Skills Data Pool’

	1. Active Electronic Games and Motor Skills (*GamesSkill*) [30, 31]	2.Global Assessment of Children’s Motor Competence (*Global_MC)* [25]	3. Fitness, Activity, and Skills Testing (*FAST*) [26]	4. Healthy Active Preschool Years (*HAPPY*) [27, 34, 35]	5.The Infant Feeding Activity and Nutrition Trial (*InFANT*) [28, 36]	6.Physical Education and Physical Literacy (*PEPL*) [29]	7. Actual and Perceived Skill and Physical Activity (*SkillPA*) [23, 24]	8.Supporting Children’s Outcomes using Rewards, Exercise and Skills (*SCORES*) [32, 33, 37]
Location	VIC	VIC	VIC	VIC	VIC	VIC	VIC	NSW
Design	Intervention	Longitudinal (two waves)	Cross-sectional	Longitudinal (three waves)	Intervention study (three waves of follow-up)	Intervention study	Cross-sectional	Intervention
Time-point used	Baseline	Baseline	Baseline	Baseline	Third follow-up	Baseline	Baseline	Baseline
Sampling strategy	Convenience sampling in schools	Convenience sampling in schools	Random sampling of schools	Randomly selected childcare/pre-schools	Randomly selected first time parent groups	Purposive sampling	Convenience sampling in schools	Purposive sampling
N consented (% consent rate)	119 (23.3%)	Australia: 144 (22.6%)	138 (30.1%)	1032 (11%)	542 (86%)	297 (91%)	136 (44.2%)	460 (78%)
Age group targeted	4–8 years	7–9 years	8–11 years	3–5 years	5 years^A^	9–11 years	5–8 years	7–10 years
Data collection dates	Apr-Jul 2014	Jul-Aug 2016	Jul-Nov 2014	May- Nov 2009	Feb-Oct 2013	Feb 2013	Aug 2013	Feb-Mar 2012
Accelerometer	ActiGraph GT3X	ActiGraph GT3X+	ActiGraph GT3X+	ActiGraph GT1M	ActiGraph GT1M	ActiGraph wGT3X-BT	ActiGraph GT3X+	ActiGraph GT3X+
TGMD Version	Version 2	Version 3	Version 2	Version 2	Version 2	Version 2	Version 2	Version 2
Assessment	Live	Recorded	Live	Live	Recorded	Live	Live	Recorded

Some of these studies include measurements taken at multiple time points for the same individual (i.e., longitudinal and intervention studies). Generally, baseline measurements were used, with the exception of Study 5 (*InFANT* intervention) in which motor skill data were only collected at later time points. Even though Study 5 (*InFANT*) was an intervention, there was no effect on physical activity [38] and no differences in motor skills between the intervention and control group at this time point [39]. For Study 4 (*HAPPY*), the motor skill assessment was part of a smaller sub-study [34, 35] (hence the smaller numbers of motor skill data compared to number of children in the study – see Supplementary File 2, Table S2). Supplementary Table S2 shows the number of participants in each study with information on each demographic variable. All studies collected information on age and sex of the children, country of birth of the parents, and highest level of education. Study 4 (*HAPPY*) did not collect whether English was the main language spoken at home, Studies 1 and 7 (*GamesSkill*; *SkillPA*) did not collect the parent sex, and Study 8 (*SCORES*) did not collect parent employment. Participants from the pooled studies were included in the main analysis sample (*n* = 988) if they had data for one or both of the TGMD domains, three valid days (any) of accelerometry data, and were not missing data for sex, age, parent’s highest level of education or cultural diversity.

A variable was created to represent cultural diversity. This was defined as speaking a language other than English at home and/or the responding parent/guardian having been born in a country other than the following English-speaking countries: Australia, Canada, Republic of Ireland, New Zealand, South Africa, United Kingdom (England, Scotland, Wales, Northern Ireland) and the United States of America. This list of countries were taken from the Australian Bureau of Statistics, i.e. that culturally and linguistically diverse refers to people born overseas in countries not classified by the ABS as ‘main English speaking countries’ [40]. For Study 4 (*HAPPY*), the cultural diversity variable was only based on parent country of birth since the language variable was not collected. Parent level of education was classified into low (some high school), mid (completed high school/trade/certificate), or high (tertiary educated) and used as a measure of socio-economic status.

### Accelerometry

Physical activity was assessed using different models of ActiGraph accelerometers (GT1M, GT3X, GT3X+; ActiGraph, LLC, Fort Walton Beach, FL; Table 1). Previous research has demonstrated the acceptability of pooling different ActiGraph models to assess physical activity as there was strong agreement between devices when analysing count data collected using the vertical axis [41]. Participants were asked to wear the accelerometer on their hip during waking hours for 7–8 consecutive days, except while bathing and swimming. A customized Excel Macro and Stata were used to reduce, clean, and harmonise the 15-s epoch accelerometer data. Valid wear time was defined as at least 8 h (480 min) of total wear time on weekdays and at least 7 h (420 min) of total wear time on weekend days recorded. Non-wear time was defined as a period of consecutive zeroes equating to 20 min. For the purpose of this study, the Evenson cut-points were applied to the epoch level data and used to identify time spent in moderate- (i.e., 2296 cpm) and vigorous-intensity physical activity (i.e., 4012 cpm) [42]. These were deemed suitable since older GT1M models used in the HAPPY and InFANT studies only allow use of the vertical axis magnitude-based cut-points. In addition, these were chosen as Trost and colleagues [43] compared five sets of youth-specific ActiGraph cut-points and found that Evenson cut-points exhibited significantly better agreement across all levels of intensity than all other examined cut-points [43].

### Motor skill competence

Motor skill competence was assessed using the Test of Gross Motor Development (TGMD). The TGMD is a process-oriented assessment (i.e., testing the skill movement rather than the outcome), consisting of two subtests for locomotor and object control skills. Study 1 (*GamesSkill*) [23, 24] only collected object control and therefore does not have total TGMD scores.

Two different TGMD versions were used (2nd Edition [44] [in seven studies)] or 3rd Edition [45] [one study]), both of which are well-known, with accompanying manuals with extensive validity and reliability support [44, 45]. The two versions are generally comparable. The TGMD-3, compared to the TGMD-2, has removed the leap, added the skip to the locomotor subset, removed the underhand roll, and added the underhand throw and one-handed forehand strike to the object control subset (and renamed it ball skills).

The locomotor skills maximum subtest scores for the TGMD-2 and TGMD-3 are 48 and 46, respectively. The maximum object control skill subtest score for the TGMD-2 and TGMD-3 are 48 and 54, respectively. Each version consists of the sum of six or seven skills (e.g., run, slide, and hop for locomotor; kick and catch for object control). The total TGMD score consisted of the sum of the two subsets and ranged from 0 to 96 and 0–100 in TGMD-2 and TGMD-3, respectively, with higher scores indicating better performances.

As there are slight differences between the two TGMD versions, the merging of obtained scores was considered based on available literature and in consultation with a biostatistician, also an author. Specifically, this was informed by a study that investigated the comparability of the two TGMD versions in 270 American children followed for 2 years from Grade 3 [46]. In that study, subtest scores were converted into the percent of maximum possible scores for comparison. Authors reported that while scores were similar, the TGMD-3 scores were slightly lower [46]. The locomotor scores differed from 1.5 to 2% and equated to less than one criteria on a subtest. There was only a significant difference (described as small) in the object control/ball skill scores when children were in Grade 3 (2.7%). In the current study, our first approach to address any potential systematic differences in scores between the versions was to convert data into scaled scores as detailed in the TGMD-2 and TGMD-3 manuals [44, 45]. However, the scaling reduced the variability in scores, creating floor and ceiling effects, so these were not used in our models. Nevertheless, these are still presented for descriptive purposes in the results. Instead, we used the original TGMD-2 and TGMD-3 raw scores and included a sensitivity analysis to check for any meaningful differences in results, which is detailed in the statistical analyses section.

### Statistical analyses

Descriptive statistics for the pooled sample and each individual study were calculated as mean ± standard deviation for continuous variables and count (%) for categorical variables. Motor skill raw scores for the locomotor and object control subsets were transformed according to the TGMD-2 manual [44] so as to provide a descriptive perspective on the motor competence level for the combined sample.

A series of linear mixed models, including random intercepts for studies, were fitted to assess linear and non-linear associations between motor skill competence and physical activity. The exposure variables were object control, locomotor, and total TGMD scores. The outcome variables were moderate- or vigorous-intensity physical activity. Initial models included individual skill competence exposure and assessed linear associations with physical activity. Stata’s *margins *post-hoc command was used to produce marginal means with 95% confidence intervals following linear mixed models. Restricted cubic splines were constructed for the exposure variables with five knots located at the percentiles recommended by Harrell [47] to examine potential non-linear relationships. Models including the restricted cubic spline terms were fitted for each exposure-outcome pair, and likelihood ratio tests compared these to the corresponding linear models, with *p* < 0.05 used to determine that the non-linear restricted cubic spline model provided a better fit to the data. All models were adjusted for child age (decimal; continuous) and sex (categorical), parent education (categorical), cultural diversity status (binary), and accelerometer wear time (continuous).

Review and meta-analysis evidence suggest sex-related differences in motor skill competence (boys performing better in object control skills compared to girls [18, 48]). Therefore, further models included interactions between sex and the skill competence exposures. These included restricted cubic splines to allow for non-linear sex-specific associations for the exposure-outcome pairs which previously showed a non-linear association.

We also examined age by skill competence interactions, separately for boys and girls. Only linear associations were tested due to the smaller numbers when the data was considered in this way, along with the fact that parcelling the data into age groups (3- < 6, 6- < 9, 9+) meant some studies were reflective of only one age group bracket therefore reducing some of the advantages of the pooled dataset.

Where there was evidence of non-linear associations or interactions with sex or age group (*p* < 0.05), plots of these associations were generated and presented. All estimated physical activity accelerometer values in the plots were based on sample mode and approximate mean values for covariates – i.e., age 8 years, highest education, non-culturally diverse, and 720 min/day wear time. Interpretation of *p*-values from models was as follows: *p* ≥ 0.1 was deemed ‘no evidence’; *p* < 0.1 was deemed ‘weak evidence’; *p* < 0.05 was deemed ‘some evidence’; and *p* < 0.01 was deemed ‘strong evidence’ [49].

Sensitivity analyses were then conducted with all models rerun with i) accelerometer data based on three valid days, including one weekend (instead of any 3 days), and ii) with the sole TGMD v3 study excluded (i.e., Study 2 – *Global_MC*) to assess whether the results produced were similar.

## Results

### Descriptive results

Table 2 displays selected descriptive data for participants from the eight included studies. The pooled main sample consisted of 988 participants. There were 50.8% males. The mean age was 8 (± 2.1) years. More than half of parents had a tertiary education (60.8%), and a quarter were classified as culturally diverse (25.1%). Child mean scaled locomotor (6.3 ± 3.0) and object control (6.9 ± 3.3) skill scores can be described as ‘below average’ (values of 6–7 according to the TGMD-2 manual). The average time in physical activity per day was 40.3 ± 12.8 min in moderate- and 19.2 ± 11.2 min in vigorous-intensity physical activity.

**Table 2:** Descriptives for the participants in the main analytic sample of pooled participants (those with accelerometry (3d), TGMD, age, sex, and education data)

	Pooled sample	1.GamesSkill	2.Global_MC	3.FAST	4.HAPPY	5.InFANT	6.PEPL	7.SKILLPA	8.SCORES
	*N* = 988	*N* = 103	*N* = 119	*N* = 123	*N* = 54	*N* = 150	*N* = 133	*N* = 100	*N* = 206
Participant sex									
Male	502 (50.8%)	57 (55.3%)	79 (66.4%)	61 (49.6%)	23 (42.6%)	72 (48.0%)	62 (46.6%)	55 (55.0%)	93 (45.1%)
Female	486 (49.2%)	46 (44.7%)	40 (33.6%)	62 (50.4%)	31 (57.4%)	78 (52.0%)	71 (53.4%)	45 (45.0%)	113 (54.9%)
Participant age	8.0 ± 2.1	6.8 ± 0.9	8.5 ± 0.6	10.4 ± 0.6	4.7 ± 0.7	5.0 ± 0.1	10.4 ± 0.4	7.1 ± 0.9	9.0 ± 0.6
Parent highest level of education									
Low	94 (9.5%)	3 (2.9%)	3 (2.5%)	3 (2.4%)	3 (5.6%)	5 (3.3%)	39 (29.3%)	3 (3.0%)	35 (17.0%)
Mid	293 (29.7%)	20 (19.4%)	17 (14.3%)	24 (19.5%)	5 (9.3%)	44 (29.3%)	66 (49.6%)	20 (20.0%)	97 (47.1%)
High	601 (60.8%)	80 (77.7%)	99 (83.2%)	96 (78.0%)	46 (85.2%)	101 (67.3%)	28 (21.1%)	77 (77.0%)	74 (35.9%)
Culturally diverse*	248 (25.1%)	21 (20.4%)	24 (20.2%)	23 (18.7%)	9 (16.7%)	13 (8.7%)	92 (69.2%)	36 (36.0%)	30 (14.6%)
TGMD: Total score	61.1 ± 14.9		72.6 ± 9.5	75.4 ± 9.5	56.4 ± 13.6	48.2 ± 9.0	71.2 ± 9.1	61.7 ± 10.8	48.4 ± 9.1
TGMD: Locomotor (raw total)	30.6 ± 7.0		32.8 ± 5.0	37.4 ± 5.9	29.7 ± 7.7	26.0 ± 5.6	34.6 ± 4.8	31.7 ± 5.1	25.4 ± 5.4
TGMD: Locomotor (scaled score)	6.3 ± 3.0		8.2 ± 2.2	6.8 ± 2.7	10.3 ± 2.4	8.2 ± 1.8	5.5 ± 1.8	6.7 ± 2.0	2.7 ± 1.4
TGMD: Object control (raw total)	30.6 ± 9.5	31.7 ± 7.4	39.9 ± 7.4	37.9 ± 6.2	26.7 ± 8.7	22.2 ± 5.5	36.6 ± 6.2	30.0 ± 8.1	23.6 ± 6.4
TGMD: Object control (scaled score)	6.9 ± 3.3	8.1 ± 2.5	9.1 ± 2.5	7.5 ± 2.8	10.6 ± 3.1	8.0 ± 1.9	6.8 ± 2.4	7.0 ± 2.4	2.5 ± 1.7
Average MPA time spent across valid days (any 3 days)	40.3 ± 12.8	44.0 ± 11.2	46.3 ± 12.6	40.6 ± 12.1	38.4 ± 13.5	38.9 ± 12.4	36.6 ± 13.4	41.2 ± 12.0	38.1 ± 12.8
Average VPA time spent across valid days (any 3 days)	19.2 ± 11.2	22.3 ± 11.0	28.2 ± 14.9	22.8 ± 11.0	15.3 ± 7.4	14.2 ± 7.1	14.8 ± 8.3	20.7 ± 9.3	16.9 ± 10.4

* Defined as speaking a language other than English and home and/or the reporting parent born in country other than the following: Australia, Canada, Republic of Ireland, New Zealand, South Africa, United Kingdom (England, Scotland, Wales, Northern Ireland) and United States of America. *MPA* Moderate-intensity physical activity, *VPA* Vigorous-intensity physical activity

The smallest sample consisted of 54 children (Study 4-*HAPPY*), and the largest, 206 (Study 8-*SCORES*). The youngest participants were from studies 4 and 5 (*HAPPY* and *InFANT*), and the oldest from studies 3 (*FAST*) and 8 (*SCORES*). Studies 6 and 8 (*PEPL* and *SCORES*) had parents with lower levels of education (29.3 and 17.0% university educated). Studies 6 and 7 (*PEPL* and *SkillPA*) appeared to have more culturally diverse participants (69 and 36%). Children in Study 2 (*Global_MC*) appeared the most active (46.3 min moderate-intensity physical activity per day).

### Associations between motor skill competence and physical activity

**Fig. 1:**
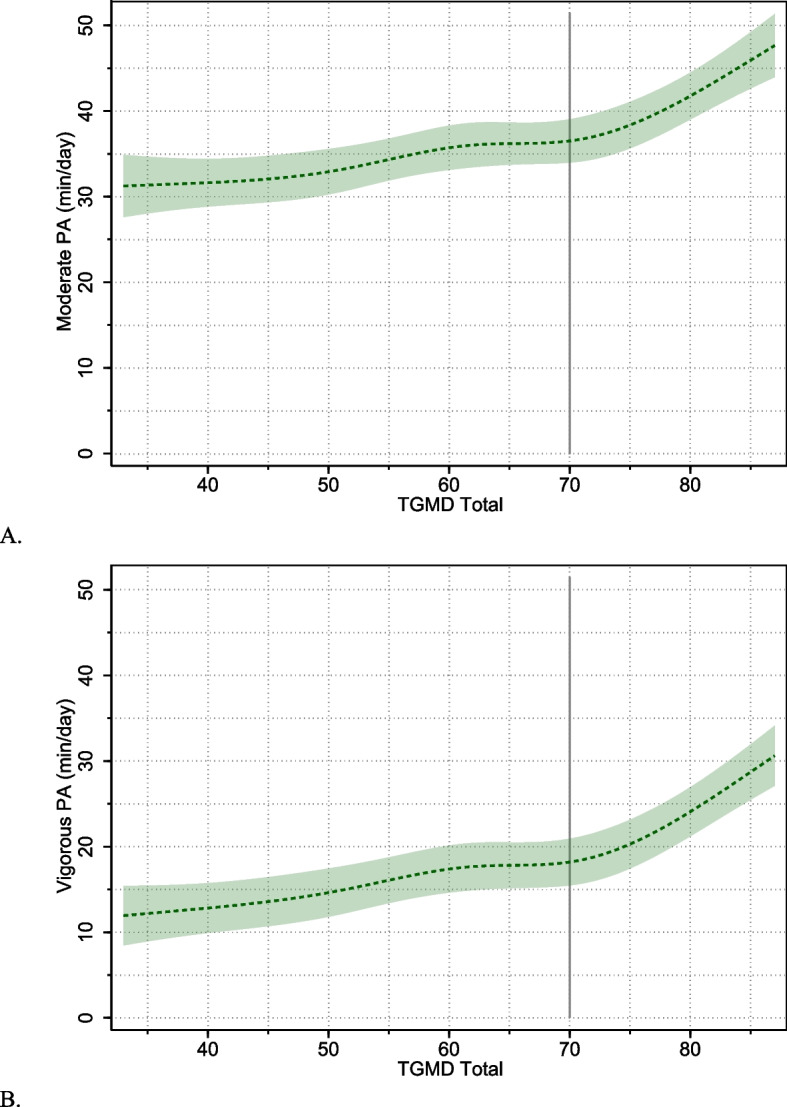
Plots of non-linear associations between TGMD total scores and moderate- and vigorous-intensity physical activity outcomes for the pooled sample. Shaded regions show 95% confidence intervals around estimated physical activity levels. The vertical line placed at TGMD total score of 70 shows the approximate point at which the strength of associations (slopes) increase noticeably

There was evidence of strong, positive linear associations between total TGMD scores and moderate- [B (95% CI) 0.27 (0.19, 0.34) *p* < 0.0005] and vigorous-intensity physical activity [B (95% CI) 0.30 (0.23, 0.37) *p* < 0.0005]; however, the restricted cubic spline models fitted better for both outcomes, indicating non-linearity in these associations (Table 3). Visual plots generated from cubic spline models (Fig. 1) indicate associations (i.e., slopes) were positive but relatively weak in the low/mid ranges of TGMD scores, but at high ranges (~ > 70/100), the associations markedly increased for both moderate- and vigorous-intensity physical activity. There was no evidence of sex interactions for total skill scores.

**Table 3:** Linear and non-linear associations between skill competence scores (measured via the TGMD) and moderate and vigorous physical activity

Outcome	Exposure	Sample (n)	Linear association			Non-linear association^a^	Sex interaction^b^
Physical activity (3 days)		988	*β*^c^	B (95% CI)	*p*-value	*p*-value	*p*-value
MPA	Total	836	0.31	0.27 (0.19, 0.34)	< 0.0005	0.001	0.14
VPA	Total	836	0.41	0.30 (0.23, 0.37)	< 0.0005	< 0.0005	0.21
MPA	Locomotor	861	0.20	0.36 (0.22, 0.50)	< 0.0005	0.19	0.19
VPA	Locomotor	861	0.28	0.43 (0.31, 0.55)	< 0.0005	0.35	< 0.0005
MPA	Object Control	963	0.26	0.35 (0.24, 0.46)	< 0.0005	< 0.0005	0.63
VPA	Object Control	963	0.33	0.39 (0.29, 0.49)	< 0.0005	< 0.0005	0.28

^a^ *p*-value for likelihood-ratio test comparing the non-linear restricted cubic spline model to the linear model. Lower *p*-values indicate more evidence that the non-linear model provides a better fit to the data than the linear model

^b^ Sex interactions were examined using linear association models for the locomotor skills exposure models, and non-linear restricted cubic spline models for the object control and total skills models as these had shown evidence (at the *p* < .05 level) of non-linearity in the overall models

^c^ Standardised linear association

*MPA* Moderate-intensity physical activity, *VPA* Vigorous-intensity physical activity

**Fig. 2:**
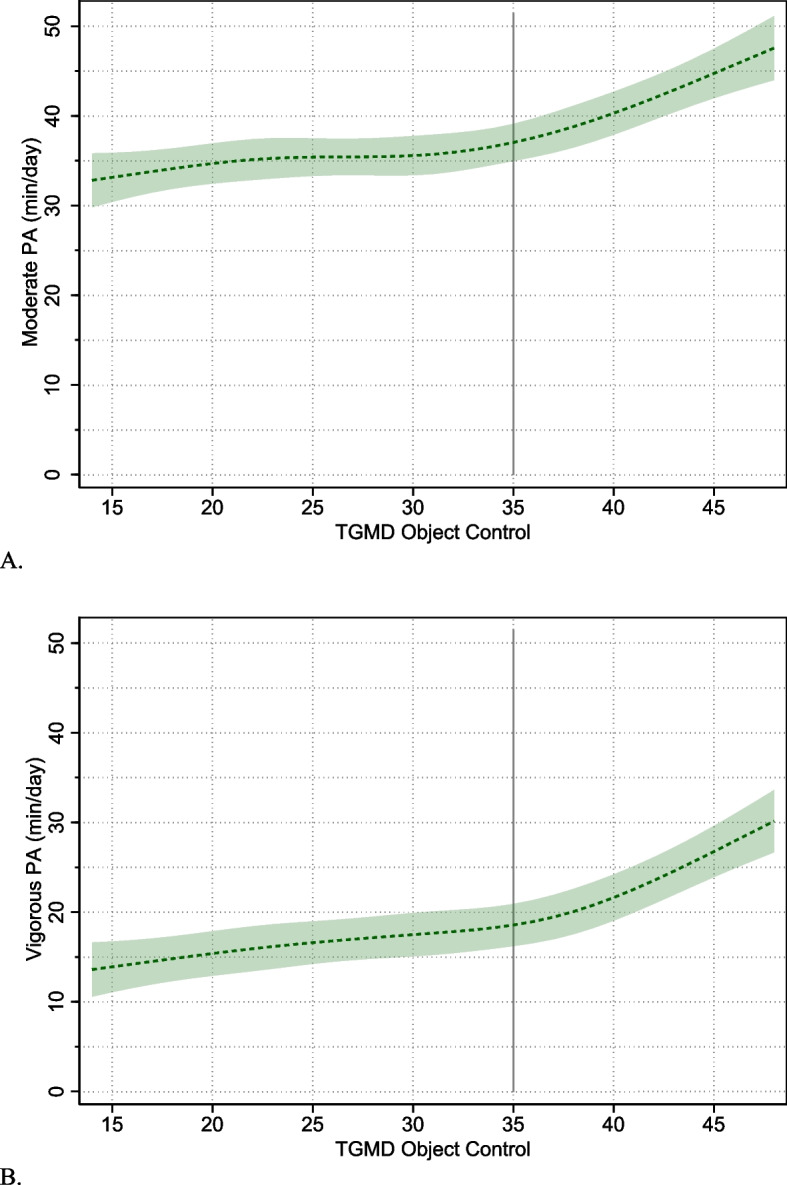
Plots of non-linear associations between TGMD object control scores and moderate- and vigorous-intensity physical activity outcomes for the pooled sample. Shaded regions show 95% confidence intervals around estimated physical activity levels. The vertical line placed at TGMD Object Control score of 35 shows the approximate point at which the strength of associations (slopes) increase noticeably

There was also evidence of strong, positive linear associations between object control skills and moderate- [B (95% CI) 0.35 (0.24, 0.46) *p* < 0.0005] and vigorous-intensity physical activity [B (95% CI) 0.39 (0.29, 0.49) *p* < 0.0005] (Table 3). However, again the superior fit of the restricted cubic spline model indicated that these associations were non-linear. Plots of these relationships showed positive but relatively weak associations in the low/mid ranges of object control scores, but at high ranges (~ > 35/50), the associations became stronger (Table 3; Fig. 2). There was no evidence of sex interactions with object control scores.

**Fig. 3:**
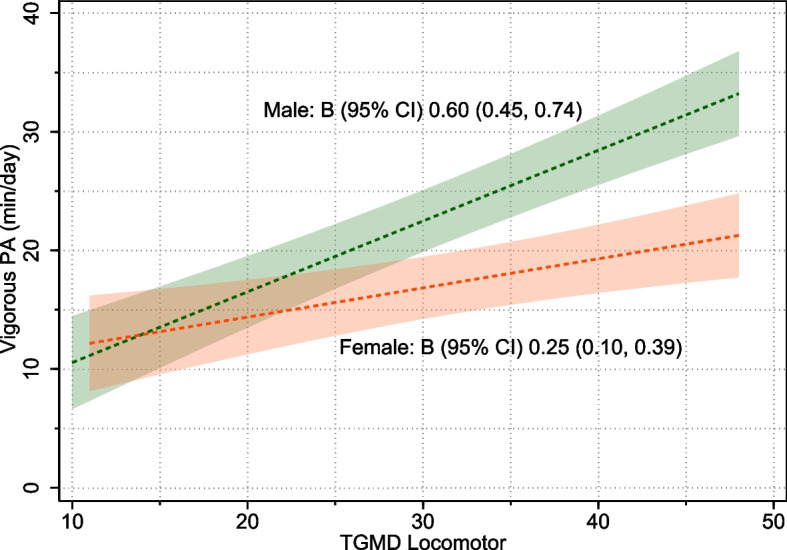
Plots of linear associations between TGMD locomotor scores and vigorous-intensity physical activity, separately by sex (sex interaction *p* < 0.0005). Shaded regions show 95% confidence intervals around estimated physical activity levels. PA: Physical activity

There was also strong evidence of positive linear associations between locomotor skills and moderate-intensity physical activity [B (95% CI) 0.36 (0.22, 0.50) *p* < 0.0005] and vigorous-intensity physical activity [B (95% CI) 0.43 (0.31, 0.55) *p* < 0.0005]. There was no evidence of non-linearity in these relationships (Table 3 and Additional File 5, Supplementary Fig. 1). There was strong evidence of a sex interaction in the association between locomotor skills and vigorous-intensity physical activity [*p* < 0.0005; Boys: B (95% CI) 0.60 (0.45, 0.74); Girls: B (95% CI) 0.25 (0.10, 0.39)], with the plot indicating boys and girls with low locomotor scores (~ < 20/50) are similar in terms of vigorous-intensity physical activity. However, males had greater increases in vigorous-intensity physical activity with increasing locomotor scores (Fig. 3). There was no evidence of sex interaction for moderate-intensity physical activity.

**Fig. 4:**
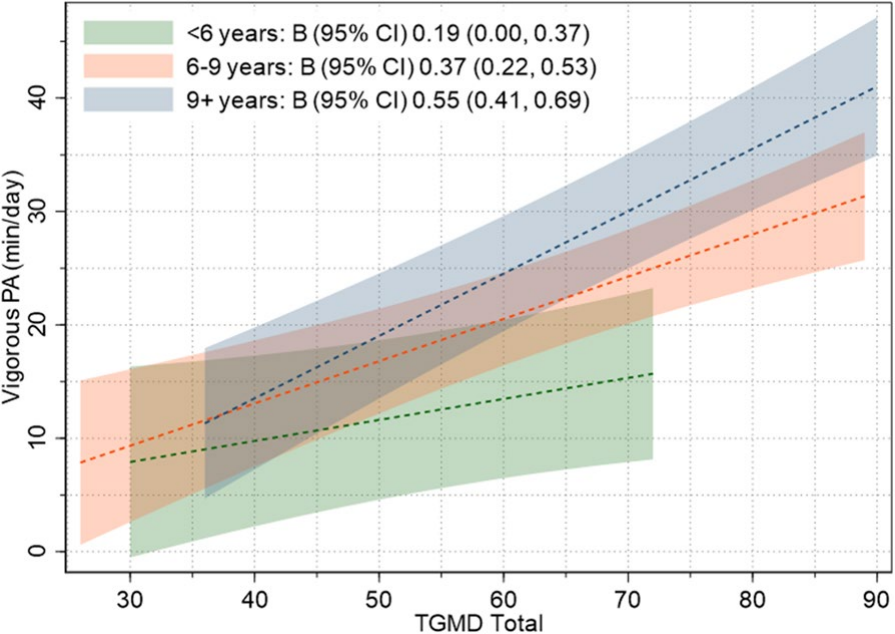
Plots of linear associations between TGMD total scores and vigorous-intensity physical activity among boys, separately by age group (age group interaction *p* < 0.0005). Shaded regions show 95% confidence intervals around estimated physical activity levels. PA: Physical activity

Among boys, there was evidence of age-group interactions for associations between both total TGMD scores (*p* = 0.006) and object control skills (*p* = 0.017), with vigorous-intensity physical activity (Fig. 4). In both instances, the interactions were driven by greater positive associations seen for boys 9 years or older compared to those younger than 6 years [TGMD total: B (95% CI) 0.36 (0.13, 0.60); object control: B (95% CI) 0.53 (0.17, 0.90)]. There was no evidence of age-group interactions for associations between motor skill competence and physical activity for girls.

### Sensitivity analyses

All models showed similar results when rerun i) with the stricter accelerometer criteria (i.e., including a weekend day) (Supplementary File 3, Table S3) and ii) without the study which used the TGMD-3 (Supplementary File 4, Table S4).

## Discussion

This study assessed the association between motor skill competence (object control and locomotor skills), and physical activity of moderate- and vigorous-intensity and considered interactions with sex. The non-linear models examining the association between total motor skills and object control skills and both moderate- and vigorous-intensity physical activity provided a better fit to the data than the linear models. This supports the hypothesis of Seedfeldt [7, 8] that once children reached a certain threshold of skill competence, associations with physical activity intensify. While our results showed a positive relationship between motor skill competence and physical activity in general, there was evidence of thresholds at which increases in motor competence were associated with even greater gains for physical activity. The thresholds corresponded to approximately 70 out of 100 for the motor competence total score and 35 out of 50 for object control skills, suggesting these skill competence thresholds as possible targets to aim for to increase children’s daily physical activity levels. It is important to reiterate that these are considered fundamental motor skills and that typically developing children should be able to achieve these skills [50]. Yet, globally, many children do not acquire these skills even by the end of primary school (around 12 years of age) [48]. Therefore, this finding provides further impetus that we need to focus on developing children’s motor skills across childhood [51] .

It is important to understand whether these relationships worked differently for boys and girls, as girls are typically less active than boys, and whilst all children experience declines in physical activity over time, girls’ activity levels decline at a greater level [52], especially during puberty. We found that the non-linear association for total skills and object control skills was the same for boys and girls, as no sex interactions were observed. Review data demonstrate that girls are typically less object control competent than boys [18, 48], and this was the case in this study also (data not shown). Therefore, it appears that developing girls’ object control skills is important from an equity perspective; as in our sample, if girls had these skills, they experienced the same physical activity levels as boys. This strategy may help to address the poor physical activity levels in girls and there is Australian evidence that these approaches can work. A RCT in early adolescent girls demonstrated that actual [53] and perceived object control skills [54] could be increased in only 12 weeks. The Australian Dads and Daughters 8 week program with girls aged 4–12 years, improved daughters’ movement skill proficiency (perceived and actual) and also, father and daughter physical activity levels [55].

This does not imply that the nature of physical activity participation will be the same for boys and girls if girls have the same level of object control skills, as girls and boys tend to have different profiles in terms of their choice of and opportunities for physical activity [56]. Perception of skill competence and how it is formed, is an important variable to consider. A recent study which using a sample from the above mentioned Dads and Daughters program, observed that girls with higher gender stereotyped attitudes had lower overall perceptions of skill, including, object control, perceptions [57].

Another complexity is considering the age group interactions. For total and object control skill there were interactions with vigorous activity, but only for boys. Younger boys had limited ranges of TGMD scores compared to the older age groups and for boys aged 9 years and older the association between total skills (and also, object control skills) and vigorous activity was stronger than for the younger boys. The interactions were not significant for girls, which may be because girls (of whatever age) had lower and generally less variable object control scores than boys.

For locomotor skills, the findings did not support the Seefeldt hypothesis [7, 8], in that a linear association better fitted the data for both moderate- and vigorous-intensity physical activity. As children become more skill competent in locomotor skills, they also become more moderately and vigorously active. It is unclear why the relationship between physical activity and locomotor skills is linear, whereas with object control skill performance there seems to be a threshold of skill that translates to higher daily physical activity. It could simply be that the ability to catch and control balls is an important skill required in many games and sports and without that fundamental skill, participation is not possible – thereby providing a threshold barrier. Past longitudinal studies support this finding in that childhood object control skill – not locomotor skill - predicted subsequent adolescent physical activity levels [58, 59].

For the locomotor skill subset, the linear relationship was different for boys and girls, with boys with higher locomotor scores engaging in more vigorous-intensity activity than girls. This implies that even when girls master these locomotor skills that they will not get to the same levels of vigorous-intensity physical activity as boys. No other studies to our knowledge have highlighted a similar finding. It is unclear why this would be the case, but likely, the reason again points to gender socialisation of girls, different interests, and opportunities. This finding does reiterate again the importance of fostering object control skill development in girls, as proficiency in object control skills in the current study, *did* lead to girls having similar physical activity levels (measured as intensity in minutes per day) as boys. Examining this relationship from the reverse perspective (i.e., with motor competence as the outcome) is also useful in understanding how to reduce sex differences. For example, one study in over 300 Portuguese children from six age cohorts (5 to 9 years of age) followed consecutively for 3 years reported that motor competence development was non-linear across time and that girls outperformed boys - once covariates such as weight status, fitness, and physical activity were included within the model [60].

Very few studies have attempted to model non-linear relationships between these variables highlighting the novelty of this work, even though studies suggest that children develop their motor competence in a non-linear fashion [60]. Typically, studies have used linear modelling to investigate whether motor skills are associated with physical activity. Exceptions include a recent American cross-sectional study which suggested evidence for a proficiency barrier. Children (9.50 ± 1.24 years) with more advanced skill levels (using the TGMD-2, 65–100 percentile) were 2.5 times more likely to meet the moderate- to vigorous-intensity physical activity guideline than lower-skilled children [61]. This finding also suggests that the relationship between motor skill and physical activity is not linear, although a non-linear relationship was not modelled. One longitudinal study over 2 years investigated the idea of a proficiency barrier using a product-oriented (i.e., outcome of the skill such as how high for a jump) assessment (Körperkoordinationstest Für Kinder [KTK]) and device-measured physical activity [62]. In this sample of ~ 700 Portuguese children aged 10 years, motor skill cut-off scores that best discriminated between less active and more active children were selected as the threshold. In the second stage (with a smaller sample of ~ 200), the odds ratio result for *low* moderate- to vigorous-intensity physical activity based on the baseline motor score classification was not significant. However, chi square results showed that if adolescents do not achieve a certain score (75 in boys and 79 in girls), the risk of being *less* active increased [62]. As highlighted in the introduction, measurement differences can make interpretation between studies problematic [15].

In the current study, motor skill competence was similarly associated with both moderate- and vigorous-intensity physical activity. A recent study in Norwegian pre-schoolers (sample ~ 1000), which used the TGMD-3 and accelerometers (analysed using 33 variables from 0 to 100 to ≥15,000 cpm), reported in contrast that across the intensity spectrum of physical activity that the strongest associations were found for high-intensity physical activity [63]. Although our estimates for vigorous-intensity activity were slightly higher than for moderate-intensity physical activity, these do not directly compare with the Norwegian study. This may be due to our decision to focus on two physical activity cut-off points to identify moderate- and vigorous-intensity, rather than smaller bands of accelerometry counts. This decision was made in order to interpret and compare our findings meaningfully alongside guidelines and previous research that also focused on higher intensity physical activity, and this also allowed us to identify individuals with different behavioural time-use profiles rather than interpret data as incremental levels of body acceleration. Nevertheless, it would also be useful to analyse acceleration data across the intensity spectrum, including lighter intensities and smaller brackets, to identify specific intervention targets in the future [64] and to detect if there is a specific threshold from which stronger associations are occurring.

The practical recommendations arising from this study are that our findings provide evidence for a tangible benchmark (particularly for object control skills) for teachers and practitioners to work towards when developing motor competence programs. If we can assist children to develop this level of object control skill, we can be more confident they will also be more moderately to vigorously physically active. For locomotor skills our findings suggest it is a case of the ‘more the better’, rather than reaching a particular threshold.

### Strengths and limitations

The main strength of this study is the large sample with cultural and educational diversity that reflect the Australian population, aiding in generalisability. The observed sample’s mean scaled locomotor and object control skill scores of ‘below average’ (values of 6–7 according to the TGMD-2 manual) are similar to previously observed global scores that were lower than what might be expected (reported as lower than average to average) from the TGMD-2 manual [48]. The other strength is the standardized measurement of motor skill measurement and harmonised analysis of device-based physical activity measurement. Other studies with similar sized samples (~ 1000) are not comparable as they use product oriented motor competence measures (e.g. using the MOBAQ-throw, catch, bounce, dribble, balance, roll, jump, run [65], and the MoMo test – based on the KTK [66], i.e. backwards walk, side to side jumping, one leg balance) [67] and self-report questionnaires for physical activity, limited in accuracy for children under 10 [68]. Finally, process assessment is important as it can guide teachers and coaches on implementing change in the skill execution to assist development [16].

These were cross-sectional analyses so we cannot infer direction of causality, even though we positioned physical activity as the outcome in analysis. One important aspect of the theoretical model authored by Stodden eta al is the developmental aspect, i.e., the distinction between the influence of physical activity on motor competence in early childhood and the reverse relationship in mid- and late childhood [4]. This was not investigated in the current analysis as we would have needed longitudinal data to truly unpack this question. Further, measurement for both constructs have limitations [16]. Devices do not capture the physical activity intensity in wheeled activities such as bike riding, scooter, and skateboarding or activities such as climbing and were removed for water-based activities. Therefore, it is plausible that the models in this current study are limited in that they do not represent the type of child who possesses these types of skills and engages in these activities. Moves are being made towards the use of accelerometers to better capture different movements (e.g., cycling [69] and motor skills [70]), and there is also interest in motor skill assessment capturing the types of skill that are not traditionally captured [71]. Furthermore, the studies did not all use the same version of the TGMD, although we did address this in our analysis.

Three studies used live coding of skills, and five used video recording, which could result in systematic differences. However, this factor (method of assessment) was checked during preliminary analysis and was not a confounder with either moderate- or vigorous-intensity physical activity (data not reported). Also, all studies reported adequate interrater reliability of their assessment regardless of whether live or assessed later by video. Whilst it is commonly thought that video is more accurate, there is also some evidence (using the TGMD-2) that live coding can be just as accurate [72]. One reliability study (using the TGMD-3) reported that whether children were assessed live or by video did not seem to affect results, although it was noted that digital records were instructed to be played only once and at a normal speed [73]. Another point to note is that we have referred to parent/guardian responder, and, in most cases, this was likely the main carer. However, in some cases, other family members responded (e.g., grandparents/uncles/aunts); hence our variable created to illustrate cultural diversity may not always relate to the main carer.

## Conclusions

The current study has provided evidence that motor skill competence (assessed using a process-oriented measure) and device-measured physical activity are associated in childhood. For locomotor skills this was a linear relationship, in that higher locomotor skill was also associated with higher moderate- and vigorous-intensity physical activity. For object control and total skills (although likely driven by the object control skills) this relationship was not linear and demonstrated that there appears to be a threshold of skill which translates to higher daily physical activity (with the caveat this is cross sectional data, and the reverse pathway could also have been investigated). The practical recommendations arising from this study are that our findings provide evidence for a tangible benchmark (particularly for object control skills) for teachers and practitioners to work towards when developing motor competence programs. If we can assist children to develop this level of object control skill, we can be more confident they will also be more physically active. As our understanding grows in this area, we will better understand the nuanced relationship between the variables, for instance, using large samples to investigate how motor skill competence predicts change in physical activity over time.

### Supplementary Information

**Additional file 1:** **Supplementary Table S1.** STROBE checklist of items that should be included in reports of observational studies.

**Additional file 2:** **Supplementary Table S2.** Number of participants per study with particular data.

**Additional file 3:** **Supplementary Table 3.** Linear and non-linear associations between skill competence scores (measured via the TGMD) and moderate and vigorous physical activity – sensitivity analysis using accelerometry data from participants with at least three valid weekdays and one valid weekend day.

**Additional file 4:** **Supplementary Table 4.** Linear and non-linear associations between skill competence scores (measured via the TGMD) and moderate and vigorous physical activity – sensitivity analysis including only the seven studies which used TGMD v3.

**Additional file 5:** **Supplementary Fig. S1.** Plots of associations between TGMD locomotor scores and moderate- and vigorous-intensity physical activity outcomes for the pooled sample.

## Data Availability

Data will not be shared as this was secondary data analysis. No datasets were generated for the current study. The authors have no permission to share the original datasets. For queries regarding the pooled dataset please contact the principal investigator of the data pooling project via email: lisa.barnett@deakin.edu.au.

## Abbreviations

FAST: Fitness, Activity, and Skills Testing
GamesSkill: Active Electronic Games and Motor Skills
Global_MC: Global Assessment of Children’s Motor Competence
HAPPY: Healthy Active Preschool and Primary Years
INFANT: Infant Feeding Activity and Nutrition Trial
KTK: Körperkoordinationstest Für Kinder
MPA: Moderate-intensity physical activity
PEPL: Physical Education and Physical Literacy
SCORES: Supporting Children’s Outcomes using Rewards, Exercise and Skills
SkillPA: Actual and Perceived Skill and Physical Activity
TGMD: Test of Gross Motor Development
VPA: Vigorous-intensity physical activity
