# Supplementary material for: Motor skill competence and moderate- and vigorous-intensity physical activity: a linear and non-linear cross-sectional analysis of eight pooled trials
Source: Int J Behav Nutr Phys Act. 2024 Feb 7;21:14. doi: 10.1186/s12966-023-01546-7 (PMC10848369; doi:10.1186/s12966-023-01546-7)
Supplement: Supplementary file 2 — Additional file 2: Supplementary Table S2. Number of participants per study with particular data. [file 12966_2023_1546_MOESM2_ESM.docx]

**Additional File 2. Supplementary Table S2. Number of participants per study with particular data**

| Studies | 1.GamesSkill | 2.Global_MC | 3.FAST | 4.HAPPY | 5.InFANT | 6.PEPL | 7.SKILLPA | 8.SCORES |
| --- | --- | --- | --- | --- | --- | --- | --- | --- |
| No. participants with accel (3d) and raw TGMD data | 107 | 121 | 126 | 56 | 150 | 281 | 102 | 259 |
| No. participants with accel (3d), TGMD, and age/sex* data | 107 | 121 | 126 | 54 | 150 | 271 | 102 | 259 |
| Of these, no. with data for the following demographic measures: |  |  |  |  |  |  |  |  |
| English main language spoken in home | 96 | 119 | 122 | 0 | 150 | 226 | 99 | 259 |
| Country of birth | 103 | 119 | 123 | 54 | 150 | 134 | 100 | 208 |
| Parent sex | 0 | 119 | 123 | 54 | 150 | 135 | 0 | 208 |
| Parent highest level of education | 103 | 119 | 123 | 54 | 150 | 133 | 100 | 206 |
| Parent employment | 101 | 119 | 123 | 54 | 145 | 135 | 99 | 0 |
|  |  |  |  |  |  |  |  |  |
| No. participants with accel (3+ days), TGMD, child age & sex, and parent CALD & education data** | 103 | 119 | 123 | 54 | 150 | 133 | 100 | 206 |
| No. participants with accel (3+ days, incl. a weekend day), TGMD, child age & sex, and parent CALD & education data | 86 | 107 | 115 | 53 | 139 | 91 | 83 | 166 |

* Noting that age and sex are key covariates and are needed to convert from raw TGMD totals to scaled scores

** Analysis sample for accelerometer 3 days and one weekend day (total n=987).
