# Supplementary material for: Motor skill competence and moderate- and vigorous-intensity physical activity: a linear and non-linear cross-sectional analysis of eight pooled trials
Source: Int J Behav Nutr Phys Act. 2024 Feb 7;21:14. doi: 10.1186/s12966-023-01546-7 (PMC10848369; doi:10.1186/s12966-023-01546-7)
Supplement: Supplementary file 3 — Additional file 3: Supplementary Table 3. Linear and non-linear associations between skill competence scores (measured via the TGMD) and moderate and vigorous physical activity – sensitivity analysis using accelerometry data from participants with at least three valid weekdays and one valid weekend day. [file 12966_2023_1546_MOESM3_ESM.docx]

**Additional File 3. Supplementary Table 3. Linear and non-linear associations between skill competence scores (measured via the TGMD) and moderate and vigorous physical activity – sensitivity analysis using accelerometry data from participants with at least three valid weekdays and one valid weekend day**

| Outcome |  | Sample | Linear association | | | Non-linear  association^a^ | Sex  interaction^b^ |
| --- | --- | --- | --- | --- | --- | --- | --- |
| Physical activity  (3 + 1 days) | Exposure | 840 | β^c^ | B (95% CI) | p-value | p-value | p-value |
| Moderate | Total | 713 | 0.32 | 0.27 (0.19, 0.35) | <0.0005 | 0.001 | 0.20 |
| Vigorous | Total | 713 | 0.43 | 0.31 (0.24, 0.38) | <0.0005 | <0.0005 | 0.47 |
| Moderate | Locomotor | 733 | 0.21 | 0.37 (0.23, 0.51) | <0.0005 | 0.23 | 0.52 |
| Vigorous | Locomotor | 733 | 0.29 | 0.45 (0.33, 0.57) | <0.0005 | 0.22 | 0.001 |
| Moderate | Object Control | 820 | 0.28 | 0.37 (0.25, 0.49) | <0.0005 | 0.002 | 0.55 |
| Vigorous | Object Control | 820 | 0.35 | 0.41 (0.30, 0.52) | <0.0005 | <0.0005 | 0.27 |

^a^ p-value for likelihood-ratio test comparing the non-linear restricted cubic spline model to the linear model. Lower p-values indicate more evidence that the non-linear model provides a better fit to the data than the linear model.

^b^ Sex interactions were examined using linear association models for the locomotor skills exposure models, and non-linear restricted cubic spline models for the object control and total skills models as these had shown evidence (at the p<.05 level) of non-linearity in the overall models.

^c^ Standardised linear association
