# Supplementary material for: Motor skill competence and moderate- and vigorous-intensity physical activity: a linear and non-linear cross-sectional analysis of eight pooled trials
Source: Int J Behav Nutr Phys Act. 2024 Feb 7;21:14. doi: 10.1186/s12966-023-01546-7 (PMC10848369; doi:10.1186/s12966-023-01546-7)
Supplement: Supplementary file 4 — Additional file 4: Supplementary Table 4. Linear and non-linear associations between skill competence scores (measured via the TGMD) and moderate and vigorous physical activity – sensitivity analysis including only the seven studies which used TGMD v3. [file 12966_2023_1546_MOESM4_ESM.docx]

**Additional File 4. Supplementary Table 4. Linear and non-linear associations between skill competence scores (measured via the TGMD) and moderate and vigorous physical activity – sensitivity analysis including only the seven studies which used TGMD v3**

| Outcome |  | Sample | Linear association | | | Non-linear  association^a^ | Sex  interaction^b^ |
| --- | --- | --- | --- | --- | --- | --- | --- |
| Physical activity  (3 days) | Exposure | 869 | β^c^ | B (95% CI) | p-value | p-value | p-value |
| Moderate | Total | 730 | 0.28 | 0.24 (0.16, 0.32) | <0.0005 | 0.001 | 0.077 |
| Vigorous | Total | 730 | 0.38 | 0.25 (0.18, 0.31) | <0.0005 | <0.0005 | 0.055 |
| Moderate | Locomotor | 746 | 0.18 | 0.32 (0.18, 0.45) | <0.0005 | 0.16 | 0.14 |
| Vigorous | Locomotor | 746 | 0.28 | 0.37 (0.26, 0.48) | <0.0005 | 0.41 | <0.0005 |
| Moderate | Object Control | 853 | 0.21 | 0.30 (0.18, 0.42) | <0.0005 | 0.001 | 0.66 |
| Vigorous | Object Control | 853 | 0.29 | 0.32 (0.23, 0.42) | <0.0005 | <0.0005 | 0.51 |

^a^ p-value for likelihood-ratio test comparing the non-linear restricted cubic spline model to the linear model. Lower p-values indicate more evidence that the non-linear model provides a better fit to the data than the linear model.

^b^ Sex interactions were examined using linear association models for the locomotor skills exposure models, and non-linear restricted cubic spline models for the object control and total skills models as these had shown evidence (at the p<.05 level) of non-linearity in the overall models.

^c^ Standardised linear association
