# Supplementary material for: Motor skill competence and moderate- and vigorous-intensity physical activity: a linear and non-linear cross-sectional analysis of eight pooled trials
Source: Int J Behav Nutr Phys Act. 2024 Feb 7;21:14. doi: 10.1186/s12966-023-01546-7 (PMC10848369; doi:10.1186/s12966-023-01546-7)
Supplement: Supplementary file 5 — Additional file 5: Supplementary Fig. S1. Plots of associations between TGMD locomotor scores and moderate- and vigorous-intensity physical activity outcomes for the pooled sample. [file 12966_2023_1546_MOESM5_ESM.docx]

**Additional File 5. Supplementary Figure S1. Plots of associations between TGMD locomotor scores and moderate- and vigorous-intensity physical activity outcomes for the pooled sample**

1.
2.

Shaded regions show 95% confidence intervals around mean physical activity levels estimated by restricted cubic spline linear mixed models, plotted between the 2.5^th^ and 97.5^th^ percentiles of TGMD locomotor scores. Note that p-values for non-linearity were p=0.19 (moderate PA) and p=0.35 (vigorous PA) respectively.
